# Antiatherosclerotic Potential of Clopidogrel: Antioxidant and Anti-Inflammatory Approaches

**DOI:** 10.1155/2013/790263

**Published:** 2013-12-26

**Authors:** Najah R. Hadi, Bassim I. Mohammad, Ihsan M. Ajeena, Hussam H. Sahib

**Affiliations:** ^1^Department of Pharmacology, College Medicine, University of Kufa, Najaf, Iraq; ^2^College of Pharmacy, University of Al-Qadissiya, Diwaniya, Iraq; ^3^Department of Physiology, College Medicine, University of Kufa, Najaf, Iraq

## Abstract

*Background*. Atherosclerosis is characterized by endothelial dysfunction, vascular inflammation, and the buildup of lipids, cholesterol, calcium, and cellular debris within the intima of the walls of large and medium size arteries. *Objective*. To evaluate the effect of clopidogrel on atherosclerosis progression. *Materials and Methods*. A total of 28 local domestic rabbits were assigned to four groups: normal control, atherogenic control, vehicle control, and clopidogrel treated. Serum triglycerides, total cholesterol, HDL-C, plasma high sensitive C-reactive protein (hsCRP), plasma malondialdehyde (MDA), and plasma reduced glutathione (GSH) were measured at the end of the experiment. Immunohistochemical of aortic atherosclerotic changes were also performed. *Results*. There was no statistically significant difference between atherogenic control group and vehicle group. Levels of lipid profile, atherogenic index, hsCRP, and MDA are increased while GSH levels were decreased in animals on atherogenic diet. Immunohistochemical analysis showed that aortic expressions of VCAM-1, MCP-1, TNF-**α**, and IL-17A were significantly increased in atherogenic control group. Histopathologic finding showed that animals on atherogenic diet have significant atherosclerotic lesion. Compared to atherogenic control group clopidogrel do not have significant effect on lipid profile. Clopidogrel significantly reduces hsCRP and MDA levels and increases GSH level. Furthermore, clopidogrel treatment significantly reduced aortic expressions parameters and the histopathologic examination of the aortic arch showed a significant reduction of atherosclerotic lesion. *Conclusions*. This study outlines how clopidogrel reduces lipid peroxidation, systemic inflammation, and aortic expression of inflammatory markers and hence reduces the progression of atherosclerosis.

## 1. Introduction

Atherosclerosis is a disease of large- and medium-sized arteries and is characterized by endothelial dysfunction, vascular inflammation, and the buildup of lipids, cholesterol, calcium, and cellular debris within the intima of the vessel wall. This buildup results in plaque formation, vascular remodelling, acute and chronic luminal obstruction, abnormalities of blood flow, and diminished oxygen supply to target organs [1]. The proposed initial step in atherogenesis is endothelial dysfunction leading to a number of compensatory responses that alter the normal vascular homeostatic properties [2]. Proinflammatory stimuli, including a diet high in saturated fat, hypercholesterolemia, obesity, hyperglycemia, insulin resistance, hypertension, and smoking, trigger the endothelial expression of adhesion molecules such as P-selectin, E-selectin, ICAM-1, and VCAM-1 which mediate the attachment of circulating monocytes and lymphocyte [3, 4]. Atherosclerotic lesions develop as a result of inflammatory stimuli, subsequent release of various cytokines, proliferation of smooth muscle cells, synthesis of connective tissue matrix, and accumulation of macrophage and lipid. Atherosclerosis is likely initiated when endothelial cells overexpress adhesion molecules in response to turbulent flow in the setting of an unfavorable serum lipid profile. Clopidogrel inhibits ADP receptors on platelets, blocking GP11b/111a complex and as a result, inhibiting platelet aggregation. Animals that were fed a pro-atherogenic diet rapidly overexpress vascular cell adhesion molecule-1 (VCAM-1) [5]. VCAM-1 expression increases recruitment of monocytes and T cells to sites of endothelial injury; subsequent release of monocyte chemoattractant protein-1 (MCP-1) by leukocytes magnifies the inflammatory cascade by recruiting additional leukocytes, activating leukocytes in the media, and causing recruitment and proliferation of smooth muscle cells [6]. However, in response to signals generated within the early plaque, monocytes adhere to the endothelium and then migrate through the endothelium and basement membrane by elaborating enzymes, including locally activated matrix metalloproteinase (MMP) that degrade the connective tissue matrix. Recruited macrophages both release additional cytokines and begin to migrate through the endothelial surface into media of the vessel. This process is further enhanced by the local release of monocytes-colony stimulating factor (M-CSF), which causes monocytic proliferation; local activation of monocytes leads to both cytokine-mediated progression of atherosclerosis and oxidation of low-density lipoprotein (LDL) [7].

Platelets serve major functions in three key aspects of atherosclerosis: atherogenesis, inflammation, and atherothrombosis [8]. Therefore, the present study was undertaken to evaluate the effect of clopidogrel on the progression of atherosclerosis.

## 2. Materials and Methods

### 2.1. Animals

A total of 28 local domestic rabbits, weighing (1.1–1.5) kg, were used in this study. All experiments were conducted in the Department of Pharmacology, College of Medicine, Qadaysia University, according to the guidelines for the Care and Use of Laboratory Animals in scientific research. The animals were placed in an animal house, in a group caging system, at controlled temperature (25 ± 2°C) and ambient humidity. Lights were maintained on a 12-h light/dark cycle. The animals had free access to water *ad libitum*.

### 2.2. Drugs

Clopidogrel (plavix, sanofi aventis, BN.F-33565, France) was dissolved in ethanol (10%) [9] and used in a dose of 20 mg/kg/day [10]. A solution of drug was freshly prepared and administered once daily orally according to body weight through stomach tube.

### 2.3. Animal Model of Atherosclerosis

Induction of atherosclerosis was carried out by feeding the rabbit an atherogenic diet (2% cholesterol (BDH Chemicals Ltd Poole England, prod 43011) enriched rabbit chow) made by addition of cholesterol powder to chow pellets for 8 weeks [11, 12].

### 2.4. Experimental Protocol

After 2 weeks of acclimatization period, the animals were randomized into 4 groups (of 7 rabbits each): normal diet control group (NC, Group I), high-cholesterol diet group which served as atherogenic control (AC, Group II), high-cholesterol diet with ethanol group (vehicle, Group III), and high-cholesterol diet with clopidogrel group (Group IV). The NC group was fed normal rabbit chow, whereas the high-cholesterol diet groups were fed a 2% high-cholesterol (atherogenic) diet. The duration of treatment was 8 weeks. At the end of the experiment, food was withheld for 16–18 hours and animals were anesthetized by ketamine (HIKMA pharmaceuticals B.N 3310) at 66 mg/kg and xylazine (alfasan B.N 1004111-07) at 6 mg/kg intramuscular [13]. The chest was opened by thoracotomy, blood sample was collected directly from the heart, and aorta was separated.

After that, the following investigations were performed:

- lipid profile including total serum cholesterol (TC), low density lipoprotein (LDL), and high density lipoprotein (HDL),
- immunohistochemistray for assessment of VCAM, TNF*α*, MCP1 and IL-17A,
- oxidation parameter including MDA and GSH,
- systemic inflammatory marker hsCRP,
- histopathological examination of the aorta for assessment of atherosclerosis.

All specimens were immediately fixed in 10% formaldehyde solution for subsequent processing.

### 2.5. Biochemical Procedures

Serum lipid profile, including total cholesterol and TG, was determined by enzymatic methods using an automatic analyzer (Abbott, Alcyon 300, USA). Plasma GSH levels was determined using methods of Beutler [14]. Plasma MDA level were determined by using competitive inhibition enzyme immunoassay technique (cusabio; Catalog no. CSB-E13712Rb). The determination of hsCRP was done by using rabbit high-sensitive CRP ELISA kit supplied by (Kamiya Biomedical Company; Cat. no. KT-097). The measurement was carried out according to the manufacturer's instructions.

### 2.6. Histological Examination of the Aorta

**Figure 1:**
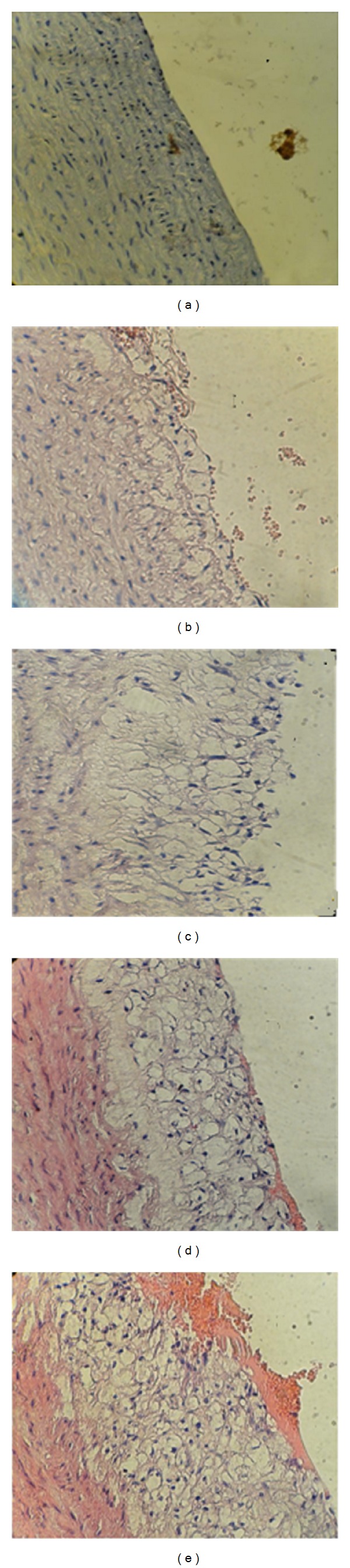
A cross section of aortic arch from hypercholesterolemic rabbit represented atherosclerosis progression (×40). (a) Normal arterial appearance, (b) initial atherosclerotic lesion characterized by lipid laden macrophage (foam cells), (c) intermediate atherosclerotic lesion characterized by extracellular lipid pool, (d) advance atherosclerotic lesion characterized by core of extracellular lipid, and (e) complicated atherosclerotic lesion characterized by haemorrhagic thrombus.

For histological evaluation of atherosclerosis, the specimens were processed in usual manner and embedded in paraffin and cut into 5 *μ*m thick sections. The tissue sections were stained with hematoxylin and eosin. The assessment of atherosclerotic changes was performed according to the American Heart Association classification of atherosclerosis: Type I and Type II lesions (early lesions), Type III lesions (intermediate lesions or preatheroma), Type IV lesions (atheroma), Type V lesions (fibroatheroma or advance lesion), and Type VI lesions (complicated lesions) [15] (Figure 1).

### 2.7. Immunohistochemistry

**Figure 2:**
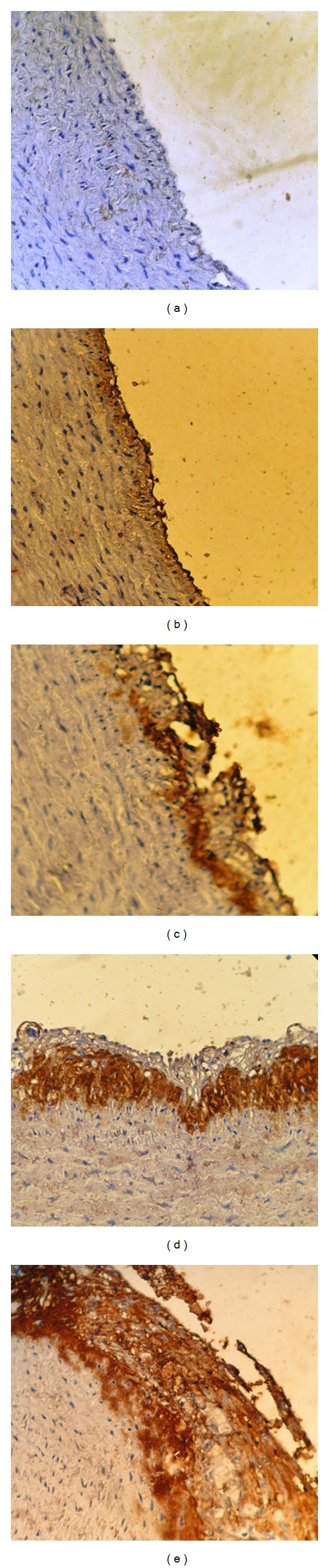
Immunohistochemical staining for MCP-1 expression in aortic arch from cholesterol-fed rabbits (×40). (a) Negative, (b) weak stain intensity, (c) moderate stain intensity, (d) strong stain intensity, and (e) very strong stain intensity.

**Figure 3:**
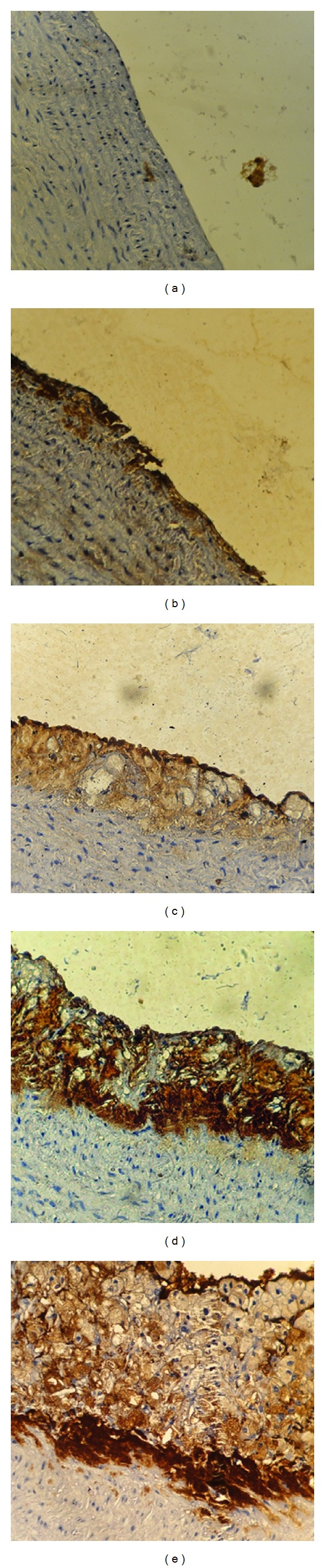
Immunohistochemical staining for MCP-1 expression in aortic arch from cholesterol-fed rabbits (×40). (a) Negative, (b) weak stain intensity, (c) moderate stain intensity, (d) strong stain intensity, and (e) very strong stain intensity.

**Figure 4:**
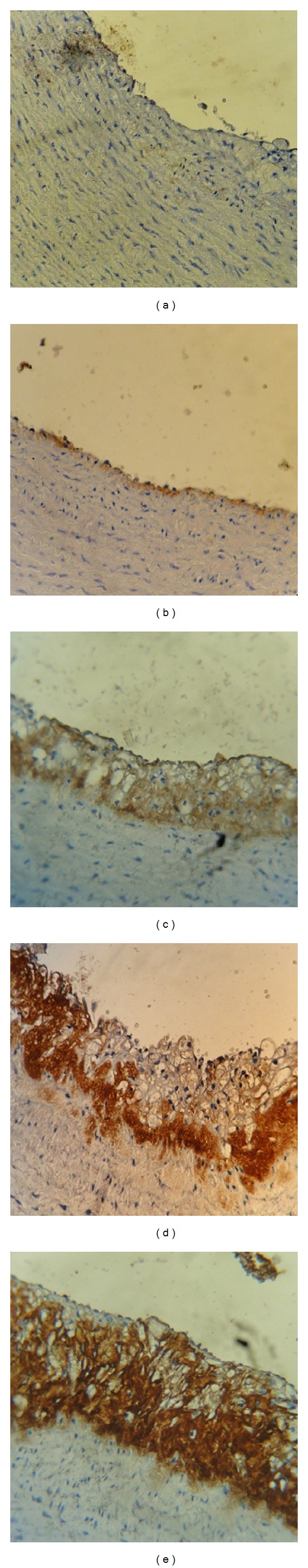
Immunohistochemical staining for TNF-*α* expression in aortas arch from cholesterol-fed rabbits (×40). (a) Negative, (b) weak stain intensity, (c) moderate stain intensity, (d) strong stain intensity, and (e) very strong stain intensity.

**Figure 5:**
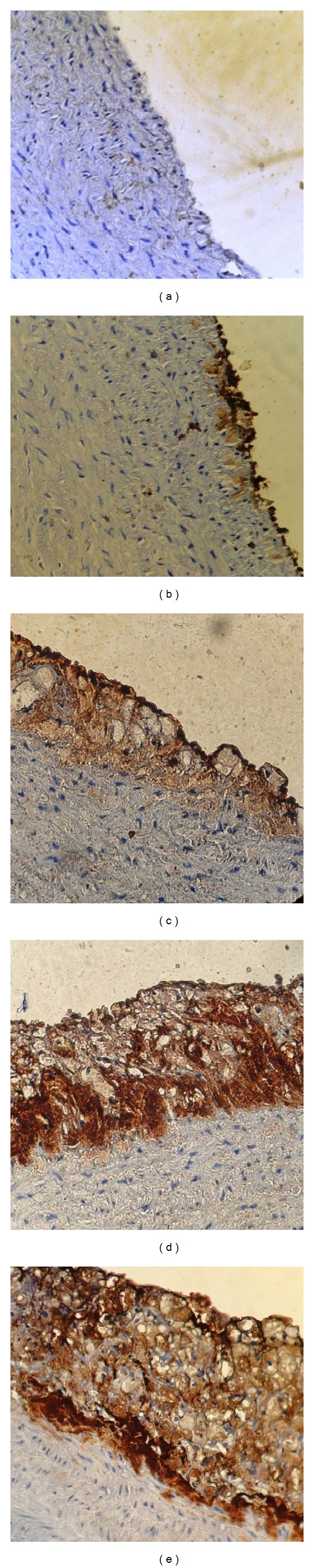
Immunohistochemical staining for IL17 expression in aortas arch from cholesterol-fed rabbits (×40). (a) Negative, (b) weak stain intensity, (c) moderate stain intensity, (d) strong stain intensity, and (e) very strong stain intensity.

It was performed with polyclonal goat antibodies and raised against rabbit VCAM-1, TNF*α*, MCP-1, and IL-17A. Staining procedure was carried out according to the manufacturer's instructions (Santa Cruz Biotechnology, Inc). The stain intensity was scored to 0: indicated no staining, 1: weak, 2: moderate, 3: strong, and 4: very strong stain intensity [16] (Figures 2, 3, 4, and 5).

### 2.8. Statistical Analysis

Statistical analyses were performed using SPSS 12.0 version. Data were expressed as mean ± SEM. Paired *t*-test was used to compare the mean values within each group at different time. Analysis of Variance (ANOVA) was used for the multiple comparison among all groups. The histopathological grading was assessed by Mann-Whitney test. In all tests, *P* < 0.05 was considered to be statistically significant.

## 3. Results

### 3.1. Effect of High-Cholesterol Diet

There was no statistically significant difference between AC group and vehicle group. Compared to NC group, the AC showed significant changes in serum lipid profile, oxidation, and inflammatory markers. Serum levels of TC, TG and LDL-C as well as plasma levels of MDA and hs-CRP were significantly (*P* < 0.001) increased. In addition plasma levels of GSH were significantly (*P* < 0.001) lower in rabbits that fed on cholesterol-enriched diet in comparison to animals on normal diet (Tables 1, 2, and 3).

### 3.2. Effects of Clopidogrel Treatment

Compared to atherogenic control, treating hyperlipidemic rabbits with clopidogrel resulted in significantly (*P* < 0.001) lower levels of plasma hs-CRP and MDA. However, clopidogrel treatment caused no significant alteration (*P* > 0.05) in the levels of serum lipids and GSH (Tables 2 and 3).

### 3.3. Immunohistochemistry

**Figure 6:**
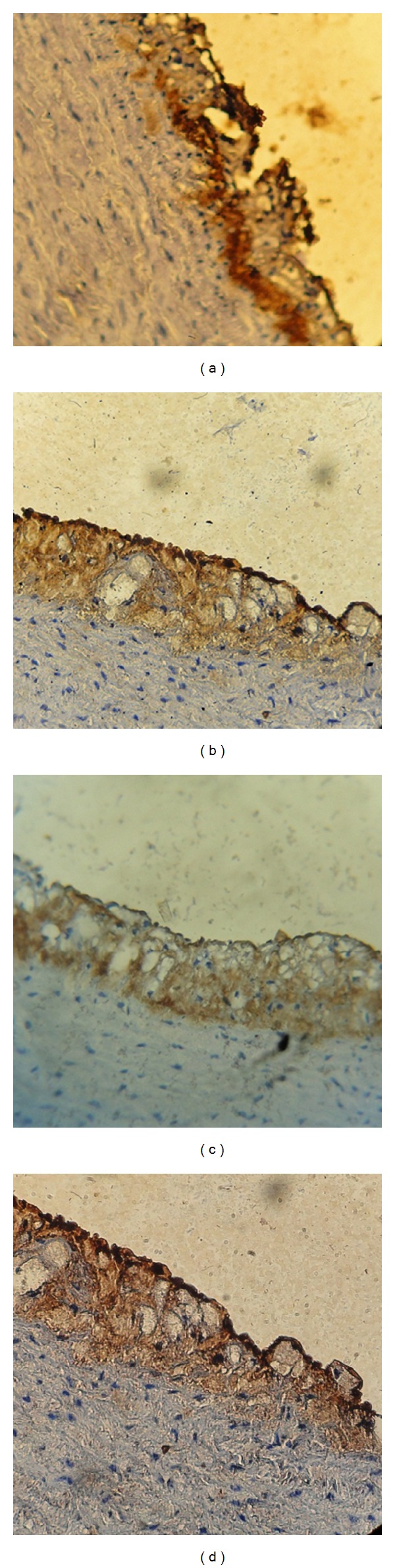
Immunohistochemical staining for VCAM, MCP-1, TNF, and IL-17 expressions in aortas arch from clopidogrel treated group (×40). (a) VCAM-1, (b) MCP-1, (c) TNF, and (d) IL-17.

The results of immunohistochemical analysis for rabbit's aortic arch of VCAM-1, MCP-1, TNF-alpha, and IL-17A were significantly different between the 4 study groups. The median intensity of these markers was the highest in AC group (very strong for all markers) and the lowest in NC group (normal for all markers). Clopidogrel treated group was associated with a median stain intensity of moderate for VCAM-1, MCP-1, TNF-alpha, and IL-17A that is significantly lower than the atherogenic control (Figure 6).

### 3.4. Histopathological Findings

**Figure 7:**
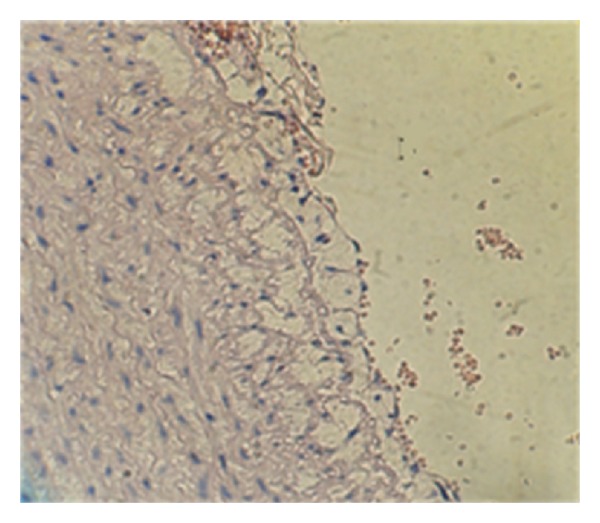
A cross section of aortic arch from clopidogrel treated rabbit represented histopathological morphology (×40).

The atherosclerotic lesions of aortic arch were graded as normal, initial, intermediated, advance, and complicated lesions (Figure 1). The median histopathological grade of atherosclerotic changes was significantly different between the 4 study groups. The median was the highest in atherogenic control (advance) and the lowest in the normal diet control (no abnormality). Clopidogrel treated group was associated with a median aortic change (initial) that is significantly lower than the atherogenic control (Figure 7).

## 4. Discussion

### 4.1. Effect of Clopidogrel on Lipid Profile

In this study clopidogrel had small nonsignificant effect on lipid profile in comparison with induced untreated study group. Such findings are consistent with that of Gu and his followers [17].

### 4.2. Effect of Clopidogrel on Oxidation Stress and Inflammatory Parameters

Like what Srinivas and his followers concluded in 2008 [18], the results showed that clopidogrel had significant effect on plasma MDA and GSH levels. Clopidogrel inhibited the increased plasma MDA level in AC group and it increased the plasma GSH level that was lowered in AC group. Furthermore, the results demonstrated a significant effect of clopidogrel on inflammatory acute phase protein by reducing the elevated hsCRP in rabbits on high fat diet. This finding suggests that the anti-inflammatory activity of clopidogrel on the vascular inflammatory responses is induced by high fat diet. This hypothesis is supported by similar findings and conclusions of other researchers [19, 20].

### 4.3. Effect of Clopidogrel on Aortic Expression of Immunohistochemistry Parameters (VCAM-1, MCP-1, TNF-*α*, and IL-17)

The significant reduction of elevated VCAM-1, MCP-1, TNF-*α*, IL-17 in atherosclerosis model of hypercholesterolemia rabbit shows the effect of clopidogrel on such inflammatory markers. This finding was also reported by Li and his followers [21]. So, clopidogrel can reduce inflammation that underlies the chronic process of atherosclerosis by reducing platelet-dependent upregulation of inflammatory and proatherothrombotic functions in leukocytes [22]. The present study is one of the very few studies that demonstrated that clopidogrel treatment significantly suppress atherosclerotic lesion induced by atherogenic diet in rabbits as compared with induced untreated group [21]. Clopidogrel will reduce expression of hsCRP and platelet-derived growth factor and also reduce intimal thickness. These results suggest that clopidogrel can retard the progression of established lesions related to inhibiting inflammation, cell proliferation, and promotion of cell apoptosis [23].

The role of IL-17A in atherosclerosis remains controversial, with different studies suggesting either a proatherogenic or an atheroprotective role. Taleb and his followers revealed that the loss of suppressor of cytokine signalling (SOCS) 3 in T cells increased both IL-17 and IL-10 production which induced an anti-inflammatory macrophage phenotype and subsequently led to unexpected IL-17-dependent reduction in lesion development and vascular inflammation [24]. On the other hand, Smith and his followers demonstrated that IL-17A plays a proatherogenic inflammatory role during atherogenesis by promoting monocyte/macrophage recruitment into the aortic wall [25]. Furthermore, Pietrowski and his followers reported that IL-17A induced an increased level of reactive oxygen species (ROS) in vascular smooth muscle cells (VSMC) [26].

We recommend further studies to use the Watanabe hereditary hyperlipidemic (WHHL) rabbit model where atherosclerosis already happened and no time is needed for induction of atherosclerosis as compared with cholesterol fed model of atherosclerosis. Furthermore, lipoprotein metabolism, atherosclerotic plaques, and coronary artery disease in WHHL rabbit model resemble those that happened in human [27]. Additionally, noninvasive imaging studies like IRON-MRI contrast imaging may be used which precisely highlight macrophage-rich plaques [28] to further clarify the effect of clopidogrel on atherosclerosis.

In conclusion, this study outlines how clopidogrel reduces lipid peroxidation, systemic inflammation, and aortic expression of inflammatory markers and hence reduces the progression of atherosclerosis.

## Figures and Tables

**Table 1:** Change in serum lipid profile in the normal control (NC), atherogenic control (AC), vehicle control (VC), and clopidogrel treated groups.

Parameters	Groups			
	Clopidogrel treated	AC	NC	VC
TC (mg/dL)	1010.30 ± 65.46^N^	1017.1 ± 64.94*	46.30 ± 0.99	1116.40 ± 42.91^N^
TG (mg/dL)	3320 ± 40.23^N^	337.10 ± 40.87*	60 ± 3.47	357 ± 35.18^N^
HDL (mg/dL)	26.10 ± 1.26^N^	24.10 ± 1.86*	15.70 ± 1.46	22.10 ± 0.77^N^
LDL (mg/dL)	917.70 ± 64.98^N^	925.60 ± 63.93*	18.60 ± 1.46	1022.90 ± 38.77^N^
VLDL (mg/dL)	66.40 ± 8.05^N^	67.40 ± 8.17*	12 ± 0.69	71.40 ± 7.04^N^

Results are expressed as mean ± SEM.

*P < 0.05, as compared to NC group.

^N^Not significant as compared to AC group.

**Table 2:** Change in mean plasma levels of hs-CRP, MDA, and GSH in normal control (NC), atherogenic control (AC), vehicle control (VC), and clopidogrel treated groups.

Parameters	Groups			
	Clopidogrel treated	AC	NC	VC
Plasma GSH (mmol/L)	0.741 ± 0.02**	0.56 ± 0.02*	1.11 ± 0.03	0.53 ± 0.01^N^
Plasma MDA (*μ*mol/L)	0.29 ± 0.01**	0.51 ± 0.01*	0.13 ± 0.01	0.51 ± 0.01^N^
Plasma hsCRP (*μ*g/L)	70.40 ± 4.19**	134.10 ± 1.20*	33.30 ± 0.78	135.70 ± 2.09^N^

Results are expressed as mean ± SEM.

*P < 0.05, as compared to NC group; **P < 0.05, as compared to AC group.

^N^Not significant as compared to AC group.

**Table 3:** The difference in median tissue (VCAM-1, MCP-1, and TNF alpha) immunostain intensity between normal control (NC), atherogenic control (AC), vehicle control (VC), and clopidogrel treated groups.

Markers	Groups			
	Clopidogrel treated	AC	NC	VC
VCAM-1	Moderate**	Very strong*	Negative	Very strong*
MCP-1	Moderate**	Very strong*	Negative	Very strong*
TNF*α*	Moderate**	Very strong*	Negative	Very strong*
IL-17 A	Moderate**	Very strong*	Negative	Very strong*

*P < 0.05, as compared to NC group.

**P < 0.05, as compared to AC group.
